# Impending myxedema as a manifestation of subclinical hypothyroidism in a case of systemic lupus erythematosus: A case report

**DOI:** 10.1097/MD.0000000000042892

**Published:** 2025-07-04

**Authors:** Ermias Habte Gebremichael, Tamirat Godebo Woyimo

**Affiliations:** a Department of Internal Medicine, Jimma University, Jimma, Oromia, Ethiopia.

**Keywords:** case report, endocrinology, levothyroxine, myxedema coma, subclinical hypothyroidism

## Abstract

**Rationale:**

Myxedema coma, the most severe form of hypothyroidism, is exceedingly rare in the setting of subclinical hypothyroidism. This case highlights the diagnostic challenge of impending myxedema coma in a patient with sepsis and systemic lupus erythematosus (SLE), despite the absence of overt hypothyroid symptoms or signs.

**Patient concerns:**

A 28-year-old female with known SLE presented to the emergency department with a 1-week history of cough, shortness of breath, pleuritic chest pain, and high-grade fever. Physical examination revealed hypotension, fever, hypoxia, pallor, oral lesions, and lung crepitations.

**Diagnoses:**

Initial investigations showed leukocytosis, elevated inflammatory markers, and imaging consistent with cryptogenic organizing pneumonia. Thyroid function tests revealed elevated thyroid-stimulating hormone with normal free tetraiodothyronine and free triiodothyronine, indicating subclinical hypothyroidism. After excluding an SLE flare and other causes, impending myxedema coma was diagnosed.

**Interventions:**

Initial treatment targeted presumed sepsis and pneumonia. Upon suspicion of impending myxedema coma, oral levothyroxine replacement therapy was initiated.

**Outcomes:**

Following levothyroxine administration, the patient’s mental status and blood pressure improved significantly. She was discharged after 2 weeks of hospitalization and remained asymptomatic at 4-month follow-up.

**Lessons:**

This case, one of only 6 reported instances of myxedema coma occurring in subclinical hypothyroidism, underscores that this life-threatening condition can develop even without classic hypothyroid features, particularly amidst significant physiological stressors like sepsis and SLE. A high index of suspicion, prompt diagnosis based on biochemical findings, and immediate initiation of thyroid hormone replacement are critical for successful management.

## 1. Introduction

Hypothyroidism is a condition characterized by an underactive thyroid gland, which fails to produce sufficient amounts of thyroid hormones. The prevalence of hypothyroidism varies globally, with an estimated 4.6% of the U.S. population aged 12 years and older affected by overt hypothyroidism, while subclinical hypothyroidism affects approximately 4.3%. This deficiency can lead to various metabolic and systemic effects, affecting multiple organ systems. Myxedema coma typically occurs in patients with long-standing untreated or poorly managed clinical hypothyroidism, and it is most commonly triggered by precipitating factors such as infections, trauma, certain medications, cerebrovascular accident, congestive heart failure, metabolic disturbances, electrolyte abnormalities, and cold exposure.^[1,2]^

Clinical manifestations of myxedema coma include profound bradycardia, hypoventilation, hyponatremia, hypoglycemia, and hypotension, often accompanied by non-pitting edema known as myxedema. Other common abnormalities seen in patients with MC include gastrointestinal dysfunction, renal impairment, hyponatremia, hypoglycemia, hypoxemia, and anemia.^[2,3]^ The diagnosis of MC is primarily clinical, with no clear-cut criteria that might distinguish either hypothyroidism alone or coma of other etiologies from true MC. Because of its high mortality of (60%) clinical diagnostic criteria were used for prompt recognition and management, including thyroid hormone replacement and supportive care to improve outcomes in these patients.^[4]^

Subclinical hypothyroidism itself is characterized by elevated thyroid-stimulating hormone (TSH) levels with normal free thyroxine (T4) levels, is generally considered to be a milder form of thyroid dysfunction and most are often asymptomatic.^[5]^ In the last decade, there have only been 5 other case reports of myxedema coma occurring in patients with subclinical hypothyroidism.^[6,7]^ Given the rarity of myxedema coma in subclinical hypothyroidism, we believe it is important to report our clinical case.

Thus, we present a 28-year-old female with a known diagnosis of systemic lupus erythematosus (SLE) under regular follow-up at our hospital, who presented with clinical futures of sepsis with pneumonia and on antibiotic management with subjective improvement of chest complains and being afebrile after antibiotics. Her clinical status deteriorated with a change in mental status and persistent hypotension which is refractory to fluid management and hydrocortisone leading to ICU admission. With further investigations after ruling out other medical causes of her deterioration. Finally, despite not having classic signs and symptoms of hypothyroidism. she was investigated with TFT leading to the diagnosis of subclinical hypothyroidism. The patient’s condition progressed despite antibiotics and steroids highlighting the need to consider impending myxedema coma and initiation of levothyroxine treatment on which she improved dramatically.

Given the uncommon nature of myxedema coma in subclinical hypothyroidism the atypical presentation of our patient lacking classic hypothyroid symptoms and its overlapping occurrence with sepsis in clinically and serologically stable cases of SLE, we believe this case warrants reporting. Understanding the intricacies of this condition, including its atypical clinical manifestations, diagnostic challenges, and management, is crucial for timely diagnosis and intervention.

## 2. Case presentation

A 28-year-old female patient who presented to our emergency department complaining of cough with shortness of breath, bilateral pleuritic chest pain, and high-grade intermittent fever, of a week duration. Associated with generalized body myalgia, blurred vision, and decreased appetite of the same period. For the past 2 years, she was diagnosed with SLE, after she presented with a history of easy fatigability, bilateral recurrent wrist joint pain, significant weight loss, and recurrent episodes of painful, self-healing perioral skin lesions. After confirmation with serological markers for SLE, she was on follow-up at the rheumatology clinic taking hydroxychloroquine 200 mg PO daily and Prednisolone tapered to 5 mg PO daily. Currently, she is in her second year of follow-up with no clinical and serologic evidence for disease activity and is adherent to her medications. Over the past 2 years of her follow-up at the rheumatology clinic, she had no similar complaints and she had no evidence of complications.

Otherwise, she had no history of orthopnoea, paroxysmal nocturnal dyspnea, abnormal body movement, body weakness, or yellowish discoloration of the skin or eyes. She had no specific symptoms of hypothyroidism, was not taking any medications other than above, and had regular menstrual cycles. She has no known personal or family history of chronic medical conditions such as diabetes, hypertension, or heart illness, and no prior history of upper respiratory tract infections, ulcers, or congestion in the nose.

**Figure 1.**
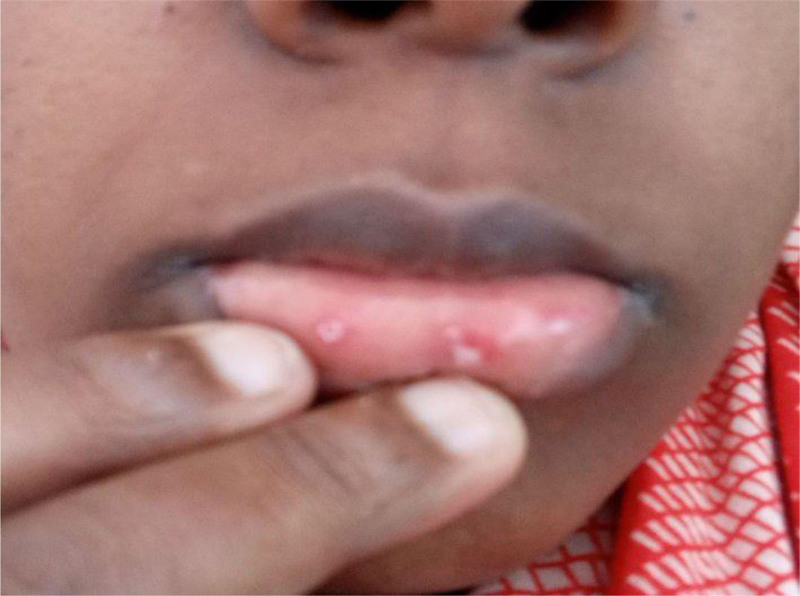
Photograph taken during admission of the patient focusing on lips; showing multiple small circular whish lesions on buccal mucosa (the picture was taken after consent taken from the patient).

Physical examination revealed blood pressure of 90/55 mm Hg (hypotensive), pulse rate of 88 beats per minute (relatively bradycardic to her hypotension), which was weak but regular, respiratory rate of 26 breaths per minute, febrile with body temperature of 37.7°C, and was desaturating with oxygen saturation of 86% with room air, later improved to 94% with intranasal oxygen support at flow rate of 2 L per minute. She was a lean female with a body weight of 48 kg, a height of 169 cm, and a body mass index of 16.80 kg/m^2^. Her conjunctivae were pale with tiny, round whitish lesions on buccal mucosae (Fig. 1). There was bilateral infra-scapular crepitation on evaluation of her lungs. The jugular venous pressure was not raised, and no abnormal findings from the precordial evaluation as well. The patient’s Glasgow Coma Scale score was 14/15 she was fully oriented in person and place and partially oriented in time; her consciousness fluctuates between being fully oriented to time, place, and person and periods of agitation, irritability, and confusion at some moments during the interview.

Complete blood count revealed leukocytosis of 15,000 white blood cells/mm^3^ with polymorphonuclear cells of 71.5% and 21.8% of lymphocytes. Highly sensitive C-reactive protein (>5 mg/L) and procalcitonin (13.51 ng/mL) were raised.

Initial diagnosis and management: At this time sepsis of chest focus with severe community-acquired multifocal pneumonia was suspected and was put on intravenous crystalloids and broad-spectrum antibiotics (1 g intravenous Meropenem administered twice a day).

**Figure 2.**
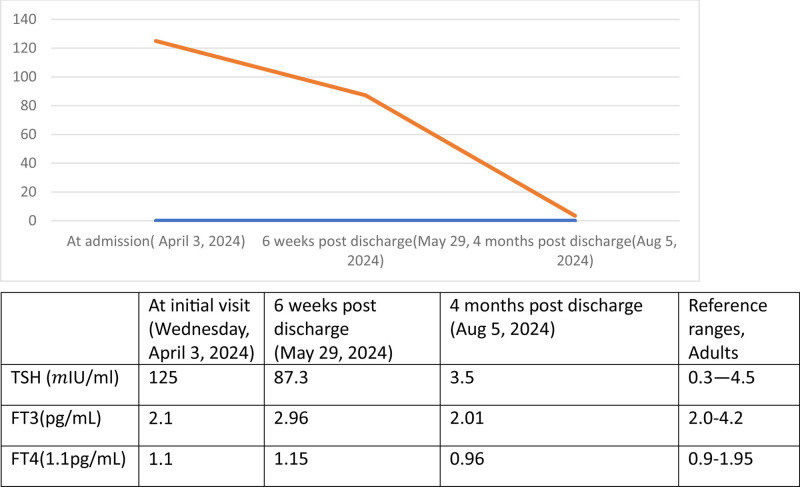
Graphic representation serial results of TSH with table blow showing details of thyroid function results which are done from admission to 4 mo post discharge of the patient. TSH = thyroid-stimulating hormone.

Diagnostic challenges: Despite the above management for 48 hours, the patient’s mental status deteriorated with a current GCS of 13/15 confused to person, place, and time, with persistent hypotension of current average Bp of 85/50 mm Hg despite aggressive fluid management. With the impression of adrenal insufficiency, she was transferred to a critical unit and started on intravenous hydrocortisone. Despite the use of vigorous intravenous crystalloids, broad-spectrum antibiotics, hydrocortisone, and critical care she had refractory hypotension and deteriorating level of consciousness. Otherwise, upon ICU revaluation, her chest complaints improved and she was afebrile. Thyroid glands were not palpable. Up on further investigations, Liver enzymes were raised suggestive of septic injury of the liver, kidney function is also deranged with a BUN of 162.6 mg/dL and serum creatine of 2.30 mg/dL, suggestive of prerenal acute kidney damage. Precontract head CT scan was unremarkable. Further investigation with thyroid function tests revealed elevated TSH of 125 (mIU/mL) with normal free triiodothyronine (Ft3 = 2.1 pg/mL) and Free Tetraiodothyronine (Ft4 = 1.1 pg/mL) (Fig. 2).

**Figure 3.**
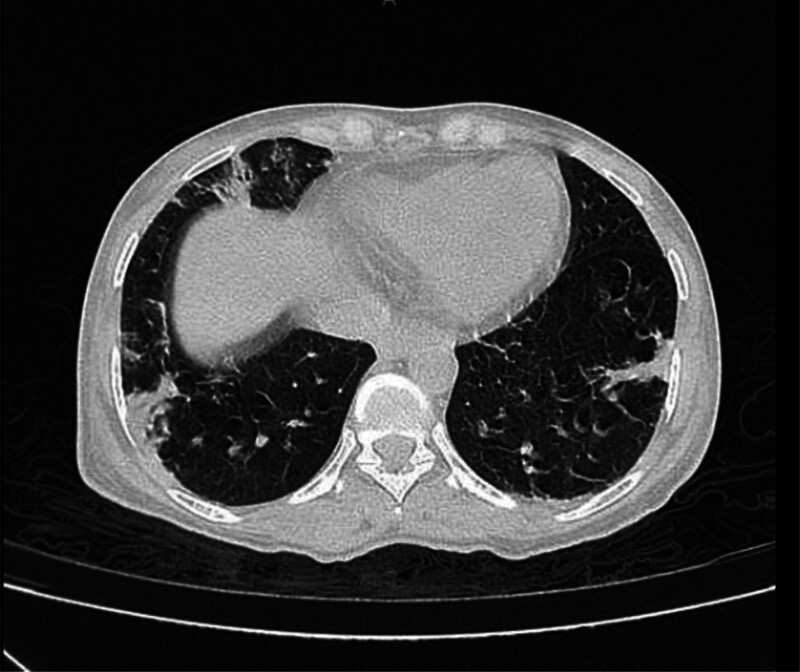
Slice-cut of a pre-contrast CT image of the lungs demonstrating cryptogenic complicated organized pneumonia. CT = computed tomography.

Anti-double-stranded deoxyribonucleic acid and ANA serologic markers were within normal range (no clinical or laboratory evidence for a flare of SLE). Bacteroides fragilis, which is susceptible to ceftazidime, vancomycin, and meropenem, was detected in the blood culture. Since she was already on meropenem we didn’t revise the antibiotics. Pre-contrast computed tomography (CT) image of the lungs demonstrating cryptogenic complicated organized pneumonia (Fig. 3 and Video 1). An Electrocardiogram of the patient showed sinus rhythm, a left axis deviation, with a rate of 120 beats per minute, and low voltage on limb and pericardial leads. Transthoracic echocardiogram and abdominal ultrasound were unremarkable. The stool Helicobacter Pylori test was negative. Pancreatic enzymes were within normal range.

Final diagnosis: Impending myxedema coma and sepsis of chest focus (improving).

Additional therapeutic intervention: she was put on a loading dose of levothyroxine 200 µg orally then 150 µg orally daily (after endocrinology team consultancy).

Follow-up and outcomes: Overall clinical status including her mental status and hypotension started to improve after 3 days of taking levothyroxine. She began full oral feeding, intravenous resuscitation was discontinued on her fifth day of hospital visit, Meropenem was discontinued on her seventh day of hospital visit and she was discharged after 14 days (11 days in intensive care unit) of admission and referred to rheumatology and endocrinology outpatient clinic, with the advice to maintain the same pharmacological treatment of disease-modifying antirheumatic drugs (DMARDs), hydroxychloroquine 200 mg po daily, and Prednisolone 5 mg po daily and to continue levothyroxine of 75 mcg po daily. Was appointed for follow-up with advice to be adherent to her medications. During her follow-up Thyroid function was reevaluated after 6 weeks, with results showing a slight decrement of TSH which is consistent with a subclinical hypothyroidism, with TSH (87.3 mIU/mL) and normal FT4 and FT3 values. At this visit, she had no clinical complaints and was adherent to her medications which were assessed with pill count and patient words. She was tolerating her medications with no newly identified drug adverse effects. She also resumes her regular job activity. The medical plan was to continue with the same dose of levothyroxine and advised to continue her follow-up after a month. Currently, she is on her fourth month of follow-up, with no clinical complaints, normalized TSH (3.5 mIU/mL), and exhibited no symptoms. Hydroxychloroquine was maintained at 200 mg/day and with the same dose of prednisolone and levothyroxine 5 mg/day and 75 mcg/day respectively. All other repeated investigations are within the normal limits (Table 1).

**Table 1:** Showing serological and urine investigations done during admission to emergency department and at fourth month post discharge follow-up of the patient.

Variables	Results on admission to emergency department*	Results at fourth month post discharge, this hospital*	Reference ranges, adults, this hospital†
Hematocrit (%)	33.5	32.9	36.0–46.0
Hemoglobin (mg/dL)	11.4	11.7	11–16.5
White cell count (per µL)	15,000	5000	4000–15,000
Differential count (per μL)			
Neutrophil (%)	71.5	76.8	1800–7700
Lymphocyte (%)	21.8	8.4	1000–4800
Monocytes	0.2	14.8	200–1200
Eosinophils	0.0	0.0	0–900
Basophils	0.0	0.0	0–300
Platelet (per µL)	240,000	249,000	150,000–450,000
Mean corpuscular volume (fL)	87.1	83	82–100
Mean corpuscular hemoglobin (pg)	32.4	33.0	26.0–34.0
Mean corpuscular hemoglobin concentration (g/dL)	35.4	36.2	31.0–37.0
Red-cell distribution width (%)	11.2	15.5	11.5–14.5
ESR (mm/h)	62	35	0–20
Creatinine (mg/dL)	2.30	0.93	0–5–1.2
Blood urea nitrogen (mg/dL)	162.6	7.6	7–20
Sodium (mmol/L)	135	142	135–145
Potassium (mmol/L)	3.18	4.9	3.5–5.5
Chloride (mmol/L)	100	97.3	98–107
Calcium (mg/dL)	8.401	9.1	8.5–10.5
AST (IU/L)	143	22.6	0–40
ALT (IU/L)	37	36.47	0–41
ALP (IU/L)	195	124.66	40–130
LDH (U/L)	759	202	140–280
Serum bilirubin (mg/dL) total	1.6	0.41	0.3–1.2
Serum bilirubin (mg/dL) direct	0.5	0.065	0.0–0.2
Random blood sugar (mm/g/dL)	220	114.26	70–110
Reticulocyte count (%)	1.9	2.1	0.7–2.5
Prothrombin time (s)	10.1	10.9	10–14
APTT (s)	100.2	29.3	22–38
INR	0.84	0.9	0.7–1.2
Hormonal analysis			
PRL (µ/L)	5.72		3.34–26.72
Blood cultures for aerobic, anaerobic, and fungal organisms	Positive for Bacteroides fragilis – which is sensitive for ceftazidime, vancomycin, meropenem	Negative	Sterile
Immunoassay and serology			
hsCRP (mg/L)	>5	0.3	0–1
Complement 3 (g/L)	0.73		0.9–1.8
Complement 4 (g/L)	0.149		0.1–0.4
Procalcitonin (ng/mL)	13.51	0.108	<0.1
Rheumatoid factor quantitative (U/mL)	<20		<20
ASO titer	Nonreactive		Negative
ANA quantitative (AU/mL)	21		0–40
Anti-dsDNA (AU/mL)	12		0–20
HIV test	Nonreactive		Nonreactive
Glycated hemoglobin (%)	5.6		4.3–5.6
Vit-B12 (pg/mL)	512		
Serum folate (ng/mL)	>20		
Direct Coombs test	Negative		
Serum VDRL test	Nonreactive		Nonreactive
Hepatitis B surface antigen test	Negative		Negative
Hepatitis C antibody test	Negative		Negative
Urinalysis	Microscopic many RBCs casts + dipstick protein + 2		
	Others are negative		
Urine LAM	Negative		
Urine culture	No growth over 14 days		
Stool *H Pylori* test	Negative		
Serum albumin (g/dL)	4.1	3.3–5.0	
Peripheral morphology			

The peripheral morphology findings are unremarkable or are normal.

ALP = alkaline phosphatase, ANA = antinuclear antibody, APTT = activated partial thromboplastin time, ASO = anti-streptolysin O, AST/ALT = aspartate transaminase/alanine transaminase, dsDNA = double-stranded deoxyribonucleic acid, ESR = erythrocyte sedimentation rate, HIV = human immunodeficiency virus, hsCRP = high-sensitivity, C-reactive protein, INR = international normalized ratio, LAM = lipoarabinomannan, LDH = lactate dehydrogenase, PRL = prolactin, TSH = thyroid-stimulating hormone, Urine LAM = lipoarabinomannan (tests based on the detection of mycobacterial LAM antigen in urine a potential point-of-care tests for tuberculosis among immunocompromised patients), VDRL = venereal disease research laboratory.

* To convert the values for urea nitrogen to millimoles per liter, multiply by 0.357. To convert the values for creatinine to micromoles per liter, multiply by 88.4. To convert the values for glucose to millimoles per liter, multiply by 0.05551. To convert the values for calcium to millimoles per liter, multiply by 0.250. To convert the values for cholesterol to millimoles per liter, multiply by 0.02586. To convert the values for triglycerides to millimoles per liter, multiply by 0.01129.

† Reference values are affected by many variables, including the patient population and the laboratory methods used. The ranges used at Jimma University Tertiary Hospital are for adults who are not pregnant and do not have medical conditions that could affect the results. They may therefore not be appropriate for all patients.

### 2.1. Strengths and limitations

The primary strength of this case report lies in its documentation of an extremely rare occurrence of impending myxedema coma in the setting of subclinical hypothyroidism, particularly in a patient with SLE presenting with sepsis and pneumonia. This adds valuable insight into the complexities of early diagnosing and managing myxedema coma, especially when traditional symptoms of hypothyroidism are absent. The report underscores the importance of maintaining a high index of suspicion and the necessity of early thyroid hormone replacement therapy in similar clinical scenarios, which could be life-saving. However, the limitations include the single-patient focus, which may not be generalizable to all cases of subclinical hypothyroidism.

## 3. Discussion

This case report highlights the diagnostic challenges faced when impending myxedema coma presents in a patient with SLE, particularly when the hypothyroidism is subclinical and complicated by sepsis. The initial presentation with symptoms such as cough, shortness of breath, chest pain, and fever raised concerns primarily for sepsis with a pulmonary focus, which is common in SLE patients due to their immunocompromised state. Sepsis, with its associated systemic inflammatory response, can mask the subtle signs of hypothyroidism, making the diagnosis of impending myxedema coma particularly difficult.^[8,9]^

A relevant feature of this case is the association between sepsis and subclinical hypothyroidism, the presence of sepsis, with rare previous cases described in the literature. With its associated inflammatory response and hemodynamic instability, can further exacerbate the effects of hypothyroidism, leading to a rapid deterioration in clinical status.^[10]^

Sepsis and myxedema coma share overlapping clinical features, including altered mental status, hypotension, and renal failure, which can lead clinicians to focus on treating the more apparent and acute condition of sepsis while the underlying thyroid dysfunction goes unnoticed. In this case, after therapy with broad spectrum and appropriate antibiotics, steroids, and vigorous fluid resuscitation, the patient became afebrile and improved her chest complaints. However, deterioration of her mental status and persistent hypotension with no clinical and laboratory evidence showing disease activity of SLE flare, it was an important decision to have an additional differential diagnosis and include the thyroid function in the assessment, even in the absence of signs or symptoms of hypothyroidism.^[10]^

The patient’s thyroid function tests revealed an elevated TSH of 125 mIU/mL with normal free T3 and T4 levels, consistent with subclinical hypothyroidism.^[10]^ However, the clinical deterioration, including persistent hypotension and altered mental status, indicated that the patient was progressing toward myxedema coma, despite the lack of overt biochemical hypothyroidism.^[10]^

Another interesting feature of this case relates to the fact that the patient did not present with physical symptoms or signs of hypothyroidism, which may happen in almost 40% and 30% of cases, respectively.^[10]^ While myxedema coma is predominantly associated with clinical hypothyroidism, its occurrence in subclinical hypothyroidism is exceedingly rare. Subclinical hypothyroidism itself is generally considered to be a milder form of thyroid dysfunction and most are often asymptomatic.^[5]^ The classic presentation of myxedema coma, including hypothermia, bradycardia, hypotension, and altered mental status, was not fully evident in this patient due to the overshadowing symptoms of sepsis. This underscores the importance of considering hypothyroidism in the differential diagnosis of critically ill patients, even in the absence of classic symptoms.

Moreover, the patient’s underlying SLE further complicated the clinical picture. SLE itself is associated with a wide range of systemic manifestations that can mimic or obscure other conditions. In this case, the chronic inflammatory state associated with SLE, combined with the use of immunosuppressive therapy (hydroxychloroquine and low-dose prednisolone), may have contributed to the delayed recognition of hypothyroidism.^[11]^

Patients with a high suspicion of myxedema coma should be treated immediately. The subsequent improvement in the patient’s clinical status following the initiation of levothyroxine therapy supports the diagnosis of impending myxedema coma. The gradual normalization of TSH levels over the follow-up period and the stabilization of the patient’s clinical condition further confirm the role of thyroid dysfunction in her acute presentation.

This case highlights the diagnostic challenge posed by impending myxedema coma in a patient with underlying SLE presenting with futures of sepsis. While the patient presented with classic symptoms of sepsis, including fever, hypotension, and altered mental status, the underlying hypothyroidism was initially masked by the severity of the infectious process.

The case also emphasizes the critical importance of considering thyroid function abnormalities in patients with unexplained hypotension, and altered mental status, especially in those with underlying conditions such as SLE or sepsis. Early recognition and treatment of impending myxedema coma are crucial for improving patient outcomes.

## 4. Conclusion

This case underscores the diagnostic dilemma posed by impending myxedema coma in the setting of subclinical hypothyroidism and sepsis in a patient with SLE. Despite the use of antibiotics and vigorous resuscitation with intravenous crystalloids and steroids her persistent symptoms of hypotension, depressed level of consciousness, and relative bradycardia dramatically improved after the initiation of levothyroxine. This case emphasizes the need for a high index of suspicion for impending myxedema coma as a differential diagnosis of subclinical hypothyroidism presenting with atypical or refractory symptoms, even in the absence of classic symptoms of hypothyroidism, particularly in the presence of sepsis or other stressors.

## Acknowledgments

The authors extend their gratitude to this patient for his cooperation in this study.

## Author contributions

**Conceptualization:** Ermias Habte Gebremichael, Tamirat Godebo Woyimo.

**Data curation:** Ermias Habte Gebremichael, Tamirat Godebo Woyimo.

**Formal analysis:** Ermias Habte Gebremichael, Tamirat Godebo Woyimo.

**Investigation:** Ermias Habte Gebremichael, Tamirat Godebo Woyimo.

**Methodology:** Ermias Habte Gebremichael, Tamirat Godebo Woyimo.

**Resources:** Ermias Habte Gebremichael, Tamirat Godebo Woyimo.

**Software:** Ermias Habte Gebremichael, Tamirat Godebo Woyimo.

**Supervision:** Ermias Habte Gebremichael, Tamirat Godebo Woyimo.

**Validation:** Ermias Habte Gebremichael, Tamirat Godebo Woyimo.

**Visualization:** Tamirat Godebo Woyimo.

**Writing – original draft:** Ermias Habte Gebremichael, Tamirat Godebo Woyimo.

**Writing – review & editing:** Ermias Habte Gebremichael, Tamirat Godebo Woyimo.

## References

[1] Klubo-Gwiezdzinska J Wartofsky L. Thyroid emergencies. Med Clin North Am. 2012;96:385–403. 22443982 10.1016/j.mcna.2012.01.015

[2] Rodríguez I Fluiters E Perez-Mendez L Luna R Páramo C García-Mayor RV. Factors associated with mortality of patients with myxoedema coma: a prospective study in 11 cases treated in a single. J Endocrinol. 2004;180:50. 10.1677/joe.0.1800347 14765987

[3] Dutta P Bhansali A Masoodi SR Bhadada S Sharma N Rajput R. Predictors of outcome in myxoedema coma: a study from a tertiary care center. Crit Care. 2008;12:1–8. 10.1186/cc6211 PMC2374608 18173846

[4] Abbey EJ McGready J Ferrucci L Simonsick EM Mammen JS. Thyroid hormone supplementation and all-cause mortality in community-dwelling older adults: results from the Baltimore longitudinal study of aging. J Am Geriatr Soc. 2021;69:1283–90. 33418603 10.1111/jgs.17015 PMC8265277

[5] Biondi B Cooper DS. The clinical significance of subclinical thyroid dysfunction. Endocr Rev. 2008;29:76–131. 17991805 10.1210/er.2006-0043

[6] Mallipedhi A Vali H Okosieme O. Myxedema coma in a patient with subclinical hypothyroidism. Thyroid. 2011;21:87–9. 21058937 10.1089/thy.2010.0175

[7] Russo R Brume E Shawkat A Dean R. Subclinical hypothyroidism with myxedema symptoms. In: A46. Critical Care Case Reports: Acid Base, Electrolytes, Endocrine, Metabolic and Renal. American Thoracic Society; 2019. p. A1711.

[8] Rittmaster RS. Antiandrogen treatment of polycystic ovary syndrome. Endocrinol Metab Clin North Am. 1999;28:409–21. 10352926 10.1016/s0889-8529(05)70077-3

[9] Mathew V Misgar RA Ghosh S. Myxedema coma: a new look into an old crisis. J Thyroid Res. 2011;2011:493462. 21941682 10.4061/2011/493462 PMC3175396

[10] Mohamed MF Mahgoub AB Sardar S Elzouki AN. Acute psychosis and concurrent rhabdomyolysis unveiling diagnosis of hypothyroidism. BMJ Case Rep. 2019;12:e231579. 10.1136/bcr-2019-231579 PMC6781965 31586959

[11] Jara LJ Vera-Lastra O Miranda JM Alcala M Alvarez-Nemegyci J. Prolactin in human systemic lupus erythematosus. Lupus. 2001;10:748–56. 11721702 10.1191/096120301717164994

